# A school-based body image intervention for young girls: is co-educational or single-sex delivery more effective?

**DOI:** 10.1186/2050-2974-2-S1-O1

**Published:** 2014-11-24

**Authors:** Candice J Dunstan, Susan J Paxton, Sian A McLean, Karen Gregg

**Affiliations:** 1 La Trobe University, Melbourne, Australia

## 

School-based body image interventions for girls have typically been evaluated in single-sex rather than co-educational settings. However, there may be advantages to including boys within classes and it might also be more practical to deliver interventions to co-educational classes. Hence, it is important to examine any difference in outcomes between these two delivery settings. This study evaluated a six-session, co-educational version of the body image intervention, Happy Being Me. Participants were Year 7 girls from 5 schools randomly allocated to receive either the intervention in a single-sex setting (n=74), co-educational setting (n=73) or no intervention control (n=53). Self-report questionnaires assessed body dissatisfaction, internalisation of media ideals, appearance comparisons, self-esteem, and depression at baseline, post-intervention, and 6-month follow-up. Improvements were found in body dissatisfaction, internalisation, appearance comparisons, and self-esteem, from baseline to post-intervention in the intervention groups compared with the control group. Intervention effects were maintained for internalisation, appearance comparisons, and self-esteem at 6-month follow-up. Baseline appearance conversations moderated body dissatisfaction outcomes. There were no significant differences in body image outcomes between single-sex or co-educational delivery formats. These findings provide further evidence of the efficacy of Happy Being Me and suggest that this intervention is equally valuable in single-sex or co-educational settings.

This abstract was presented in the **Peter Beumont Young Investigator award finalist** stream of the 2014 ANZAED Conference.

